# Right Ventricular Function in Chronic Heart Failure: From the Diagnosis to the Therapeutic Approach

**DOI:** 10.3390/jcdd7020012

**Published:** 2020-04-09

**Authors:** Francesco Monitillo, Vito Di Terlizzi, Margherita Ilaria Gioia, Roberta Barone, Dario Grande, Giuseppe Parisi, Natale Daniele Brunetti, Massimo Iacoviello

**Affiliations:** 1 Emergency Cardiology Unit, University Policlinic Hospital, 70124 Bari, Italy; fmonitillo@libero.it; 2 Cardiology Unit, Department of Medical and Surgical Sciences, University of Foggia, 71122 Foggia, Italy; vitodt89@gmail.com (V.D.T.); barone.r90@gmail.com (R.B.); dr.natale.daniele.brunetti@hotmail.it (N.D.B.); 3 Cardiology Unit, Perrino Hospital, 72100 Brindisi, Italy; m.ilariagioia@gmail.com; 4 Cardiology Unit, Sarcone Hospital, 70038 Terlizzi, Italy; dario.grande@ymail.com (D.G.); Giuseppeparisi88@libero.it (G.P.)

**Keywords:** right ventricle, heart failure, cardiac imaging, echocardiography

## Abstract

There is growing attention for the study of the right ventricle in cardiovascular disease and in particular in heart failure. In this clinical setting, right ventricle dysfunction is a significant marker of poor prognosis, regardless of the degree of left ventricular dysfunction. Novel echocardiographic methods allow for obtaining a more complete evaluation of the right ventricle anatomy and function as well as of the related abnormalities in filling pressures. Specific and effective therapies for the right ventricle dysfunction are still not well defined and this represents the most difficult and important challenge. This article focuses on available diagnostic techniques for studying right ventricle dysfunction as well as on the therapies for right ventricle dysfunction.

## 1. Introduction

Sir Harvey was the first to describe the relevance of right ventricular function and to consider the right ventricle (RV), the pulmonary circulation, and the lungs as a single unit [1]. Despite Harvey’s message and his emphasis on right heart, cardiologists traditionally focused their attention on left ventricle (LV) function, missing the importance of the RV contribution to several diseases. In the second half of the twentieth century, growing attention has been dedicated to the study and the pathophysiology of the right heart. Recently, the development of new cardiac imaging methods made the study of the anatomy, physiology, and pathophysiology of the right heart, and pulmonary circulation, more feasible. This is even more relevant if we consider that RV dysfunction plays a key role in hemodynamic and prognosis of patients affected by cardiovascular diseases and heart failure (HF) [2]. In this last clinical setting, RV failure implies an increased risk of cardiac death, regardless of LV dysfunction degree [3].

In this review, we discuss RV structure and function, its pathophysiological role in HF, and the related diagnostic and therapeutic critical issues.

## 2. Anatomy and Physiology of the Normal Right Ventricle

The RV is located anteriorly, just behind the sternum. When compared with the LV, it has a more complex three-dimensional geometry, appearing triangular when viewed from the front and it wraps around the LV [2]. This structure is the result of the combination of RV free wall transverse fibers that encompass the septum, and the oblique helical fibers of the septum [2]. The RV shape is also influenced by loading conditions, which can modify the position of the interventricular septum.

RV is coupled with a low hydraulic impedance of the pulmonary circulation and right-sided pressures are significantly lower than left-sided ones [4]. Compared with the LV, RV function is more frequently influenced by increased preload or afterload. According to the Frank–Starling mechanism, an increase in RV preload, i.e., the load at the end of diastole, can improve myocardial performance within physiological limits. Exceeding RV volume loading may lead to the compression of the LV and to global ventricular function impairment. RV performance also depends on ventricular interdependence, heart rhythm, synchrony of ventricular contraction, and RV force-interval relationship [5].

The RV is extremely sensible to changes in afterload. Slight variations in pulmonary pressure can impair RV function and global cardiac performance, even in cases of normal preload [6]. Pressure-volume loops can be helpful in better understanding the complex interactions between RV contractility, preload, and afterload. They reflect changes of pressure-volume curves in different loading conditions. The pressure-volume relationship represents the elastance, an index of ventricular contractility, and it seems to follow a time-varying elastance model. We consider the maximal elastance (Emax) as the maximum ventricular pressure to volume ratio during the cardiac cycle. Unlike the LV work curve, the normal RV pressure-volume loop has a triangular shape, probably due to low pulmonary vascular impedance. Emax is defined as the maximum ventricular wall stress or arterial hydraulic load and it occurs at the end of systole. Even if not coincident, it is assumed, for clinical practice, that Emax is equal to RV end-systolic elastance, which is the pressure to volume ratio at the end of systole [6,7].

If we consider pressure-volume loop, arterial elastance (Ea) can be obtained dividing end-systolic pressure to stroke volume. The RV function analysis must consider its relation with the afterload and it can be evaluated with the Emax to Ea ratio, which defines “right ventriculo-arterial coupling”. This concept specifically refers to the relationship between ventricular contractility and afterload, i.e., the ventricular and arterial elastances ratio. In normal conditions, the best mechanical coupling is reached when Emax/Ea ratio is 1. The normal Emax to Ea ratio for the RV is between 1.5 and 2, corresponding to the optimal energy transfer from the RV to pulmonary circulation and to the optimal balance between RV mechanical work and oxygen consumption [7,8].

## 3. Right Ventricular Dysfunction and Ventriculo-Arterial Uncoupling

RV dysfunction analysis should consider the RV and the pulmonary circulation as one. The most relevant cause of RV dysfunction is a left-sided heart disease, which can increase RV afterload and cause its dysfunction [2]. In fact, an increased LV end-diastolic pressure leads to the raise of pulmonary artery pressure, to the increase of RV afterload and, as a consequence, to worsening of RV performance even when it is not directly involved by the cardiac disease. This is due to the altered geometry of the dysfunctional LV and a disarray of the septal fibers, which become less oblique and more spherical and are responsible for the impairment of RV contractility. This process ends with a progressive RV enlargement, RV failure, and tricuspid regurgitation (TR), which imply a further reduction of the mechanical efficiency of septal fibers [9].

The chronic increase in afterload, which characterize RV dysfunction, leads RV pressure-volume loops to assume a square or rectangular shape in contrast to the typical trapezoidal shape of a mechanical efficiency/low impedance state.

**Figure 1:**
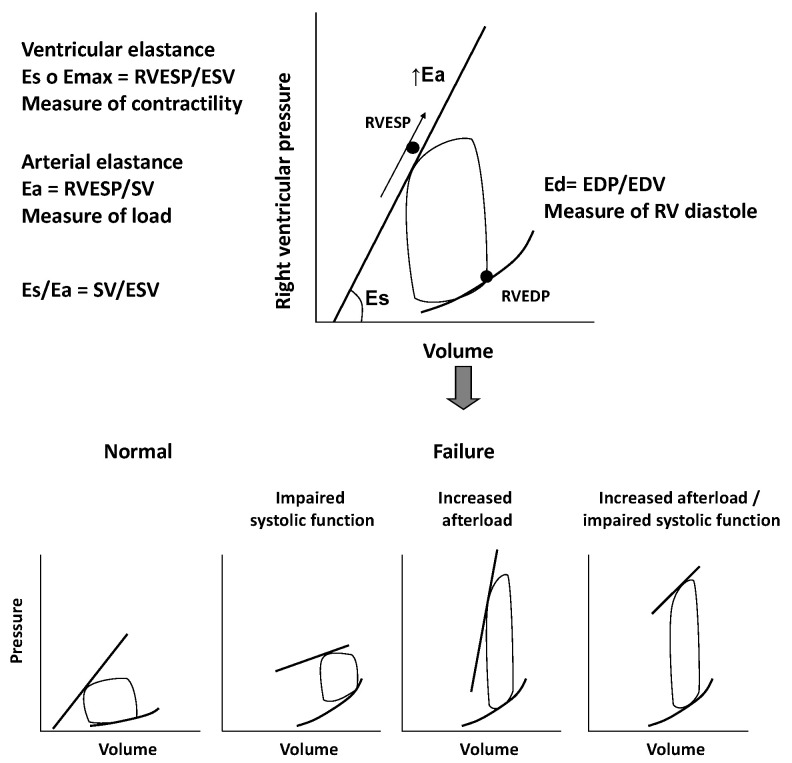
Figure shows the relation between right ventricle contractility, preload, and afterload through pressure/volume loops. In the lower part of the figure, pressure/volume loops in condition of different loading conditions and right ventricle dysfunction are displayed. Eas: End-systolic elastance; Emax: maximal elastance; RVESP: right ventricle end-systolic pressure; ESV: end-systolic volume; Ea: arteralelastance; SV: stroke volume; Ead; End-diastolic elastance; EDP; end-diastolic pressure; EDV: end-diastolic volume.

In a first compensatory phase, the response of RV to chronic overload is an increased contractility (Ees) that is proportional to the increase of Ea. This phase is characterized by ventricular hypertrophy, necessary to preserve cardiac output, at the price of increased filling pressure and diastolic dysfunction [7,8,10]. In the following transition phase, the persistence of high pressures and increased afterload lead to RV dilatation, still preserving a residual systolic function. Finally, in the failure phase, the ventricle dilates further and systolic function collapses, regardless of pulmonary afterload. At this time, RV function is irreversibly compromised and the ventricular function cannot rise despite a reduction of the pulmonary pressures. When Ees increases less than Ea, the Ees/Ea ratio decreases leading to “ventriculo-arterial uncoupling” and, therefore, to RV failure [11], as showed in Figure 1.

In HF, the early right ventriculo-arterial uncoupling is the consequence of the RV inability to adapt to the combined effects of increased pulmonary arterial resistance and elastance [8,12].

## 4. Evaluation of Right Ventricular Dysfunction in Chronic Heart Failure

### 4.1. Traditional Echocardiographic Parameters

Considering the prognostic impact of RV dysfunction in cardiovascular disease and HF, the evaluation of RV performance and structure is crucial for the clinical management of patients [3]. In clinical practice, echocardiography is the mainstay of the assessment of RV structure and function. The first step is the analysis of RV morphology and diameters from the parasternal and apical views. LV dysfunction can cause RV overload and dilatation, until the shift of the interventricular septum toward the left. LV assumes a D-shaped morphology with related impairment of RV function. The parasternal long axis and apical 4-chamber view allow to visualize the right chambers and to evaluate RV dilation by measuring the outflow and the inflow tracts main diameters [13].

Among the echocardiographic parameters, the tricuspid annular plane systolic excursion (TAPSE) is commonly used to assess the global RV systolic function. It is an M-mode measurement of systolic excursion of the RV annular segment along its longitudinal plane towards the “apex”, obtained positioning the cursor at the tricuspid annulus from a standard 4-chamber view. According to the guidelines, a value of TAPSE <16 mm indicates RV systolic dysfunction [13]. TAPSE correlates very well with other indices of RV systolic function and with the radionuclide-derived estimation of right ventricle ejection faction (RVEF) [14]. In patients affected by chronic HF (CHF), TAPSE was independently associated with a worse prognosis and provided incremental prognostic information [15,16]. In an interesting analysis of CHF outpatients with reduced left ventricular ejection fraction (LVEF), the prognostic role of a 6-month echocardiographic reassessment of RV function and of a reversible abnormal TAPSE was evaluated. The study confirmed that a reduced RV function at baseline was independently associated with poor outcome, but also that the reversibility of abnormal RV function was associated with a better prognosis, regardless of LVEF improvement, LV reverse remodeling, and cardiac resynchronization therapy [17].

Right ventricle fractional area change (RVFAC) is another index of global RV systolic function and represents the difference between RV end-diastolic and end-systolic areas fractioned RV end-diastolic area, measured by tracing the RV endocardium border at both end-systole and end-diastole in an RV focused apical view (normal value >35%). This parameter shows a good correlation with the measurement of the RVEF obtained through magnetic resonance imaging [18]. In the VALIANT (Echo) study, RVFAC was used to assess RV function after myocardial infarction and to evaluate whether it predicted a worse clinical outcome. Lower values of RVFAC were associated with an increased risk of all-cause mortality, CV death, sudden death, HF, and stroke [19].

RV myocardial performance index (MPI) or Tei index, calculated as the isovolumic time to ejection time ratio, is a parameter of the global cardiac function, providing information about both systole and diastole (normal value <0.4). As a pulsed Doppler method, it is less dependent on the quality of the images and on ventricular geometry, and it is relatively independent of the preload, the afterload, and the heart rate [20]. Tei index, when combined with the other systolic and diastolic RV functional parameters, allows to further stratify patients with symptomatic HF [21].

Tissue Doppler imaging (TDI) is an echocardiographic technique that uses Doppler principles to measure the velocity of myocardial motion. It has become an established component of the diagnostic ultrasound examination. This echocardiographic tool allows assessing both global and regional systolic and diastolic function and the timing of myocardial motion. The TDI of the RV is obtained from the apical 4-chamber view, positioning the sample volume at the tricuspid annulus or the basal segment of the free wall [22]. The TDI spectrum presents a first peak, that is the RV s’ or systolic excursion velocity, representing the velocity of the myocardial movement towards the apex (positive value). It is followed by two diastolic velocities (negative values), an early diastolic velocity (e’) and a late diastolic velocity (a’), that represent the velocities of the myocardial wall movement during the rapid early filling and the late atrial filling of the ventricle. A peak systolic velocity value (s’) less than 11.5 cm/s suggests RV systolic dysfunction, and seems to correlate with RVEF less than 45%. The related value of the RV free wall basal segment, less than 8 cm/s in patients with right ventricular infarction, is also indicative of poor prognosis. The RV demonstrated to be a reliable tool to evaluate RV systolic function, regardless of the severity of pulmonary hypertension. In a prospective analysis of patients with HF and atrial fibrillation, tricuspid e’ (<9.0 cm/s; *p* < 0.001) and septal e’ (<7.3 cm/s; *p* < 0.001) independently predicted a higher risk of cardiac events (cardiac death and readmission for HF) [23]. Similarly, in a population with heart failure with preserved LVEF and atrial fibrillation, both s’ < 5 cm/s and e’ < 7 cm/s were associated with cardiovascular death, recurrent HF, and ischemic stroke [24].

### 4.2. Strain and Strain Rate Imaging

Over the last decade, new echocardiographic parameters of RV function have been introduced. They are mainly based on the analysis of “strain”, i.e., the deformation of an object compared with its initial shape, and “strain rate” (SR), i.e., the rate of the deformation. RV strain can be obtained by TDI or speckle-tracking imaging. The extent of deformation is generally expressed as a percentage of strain compared to the initial length. For a given myocardial segment, thickening and shortening are respectively positive and negative values. Myocardial contraction implies, at the same time, wall shortening (negative values), torsion, and thickening (positive values), so all strain parameters are useful for the evaluation of the contractile function [25].

The dependence from the angle incidence of the sampling is a strong limitation of TDI. Myocardial speckle tracking, on the contrary, is angle-independent and allows studying all components of the regional and global systolic deformation with a high reproducibility (longitudinal, radial, and circumferential). However, good quality images are needed and the delineation of the endocardial border may be a potential problem for the RV evaluation [25]. From the 4-chamber view, it is possible to obtain RV global longitudinal strain (RV-GLS), which includes the septum, and the strain of only the RV free wall (RV-fwLS). RV-fwLS and RV-GLS showed a stronger correlation with the RVEF, evaluated by cardiac magnetic resonance (CMR), if compared to traditional echocardiographic parameters [26,27]. Several studies proved that two-dimension longitudinal strain allows to detect mild LV myocardial abnormalities early [28] and to stratify the prognosis of CHF outpatients [29]. Moreover, data from the literature suggest that RV strain and strain rate analysis provide a more accurate and less preload dependent estimation of the overall performance of the RV [30].

In a population of patients with advanced HF candidate to heart transplantation and evaluated with speckle-tracking echocardiography, RV-fwLS demonstrated a good correlation with RV stroke work index at right heart catheterization, as opposed to TAPSE and tricuspid s’ [31].

In a cohort of CHF outpatients, in stable clinical conditions and in conventional medical and electrical therapy, traditional echocardiographic parameters, such as TAPSE, systolic peak at TDI, and RVFAC, did not remain associated with events at multivariate analysis, whereas RV-GLS and RV-fwLS were independently associated with prognosis. RV-GLS showed a slightly greater prognostic power, probably due to the effect of systolic interventricular dependence, which greatly contributes to RV longitudinal shortening [30]. In addition, further studies have demonstrated the independent and incremental prognostic value of strain measures [32,33], even in patients who have undergone cardiac resynchronization therapy (CRT) [34], and in those with normal TAPSE [35]. These findings strengthen the clinical usefulness of these parameters in routine clinical practice.

### 4.3. Echocardiographic Measures of Right Ventriculo-Arterial Uncoupling

Pulmonary artery systolic pressure (PASP) can be estimated measuring TR transvalvular gradient, assuming it is approximately equal to the right ventricle systolic pressure (RVSP). This one can be determined combining transvalvular maximal gradient with an estimate of the right atrial pressure, obtained from the inferior vena cava diameter and its respiratory changes.

The combined assessment of RV function and PASP estimation seems to be a more accurate index of RV performance status [36]. Using echocardiography and combining TAPSE and Doppler estimated PASP, it is possible to further stratify patients through a non-invasive index of RV arterial coupling. The TAPSE/PASP ratio is an index of in vivo RV length vs. developed force and a non-invasive assay of RV contractile state [37]. It was inversely related to New York Heart Association (NYHA) functional class and the combination of the two variables was better related to prognosis [38]. A TAPSE/PASP ratio < 0.36 mm/mmHg identified patients at higher risk, irrespective of reduced or preserved ejection faction (EF). When combined with cardiopulmonary exercise testing, it provides an even more complete and relevant prognostic information. In particular, right ventriculo-arterial uncoupling, i.e., TAPSE < 16 mm and PASP ≥ 40 mmHg, combined with the peak VO2 and the highest exercise oscillatory ventilation rate, identified patients at higher risk of cardiac mortality, left ventricular assist device implantation, or heart transplant [38]. This parameter has proven to be a predictor of worse prognosis, not only in patients with HF and reduced LVEF (HFrEF), but also in those with mild reduced (HFmrEF) or preserved (FFpEF) LVEF [39].

Although TAPSE is a parameter easy to measure, reproducible, and a reliable predictor of prognosis in HF, it is not able to fully represent the complexity of RV motion. At the same time, speckle tracking derived strain parameters have demonstrated to be more accurate in the evaluation of RV function, allowing to analyze both the free wall and the interventricular septum. Therefore, the relationship between the RV contractility surrogate, assessed by two-dimensional strain, and the generated force expressed by PASP, could allow to better estimate the RV performance status. In a population of chronic HF patients, the combination of RV longitudinal strain (both RV-fwLS and RV-GLS) and estimated PASP provides a reliable index of RV-pulmonary arterial coupling and a parameter that is independently associated with an increased mortality risk and worse outcome [40].

In the evaluation of right ventriculo-arterial coupling, a last aspect should be considered. In advanced stages of RV dysfunction, the peak velocity of tricuspid regurgitation could unexpectedly be very low. In these patients, lower transtricuspid systolic gradients are the result of higher right atrial pressures and reduced RV contractility. As a consequence, the presence of low RV systolic pressure (RVSP) associated with a severe dilatation of inferior cava vein is the expression of advanced RV failure. This has been demonstrated in a cohort of 265 patients admitted for acute HF decompensation. Lower transtricuspid systolic gradient (TR gradient < 20 mmHg), lower TAPSE (<14 mm) and higher estimated right atrial pressure (eRAP) values were predictors of worse prognosis. The RV contraction pressure index (RVCPI), defined as TAPSE × TR gradient, was a parameter able to improve prognostic stratification [41].

**Figure 2:**
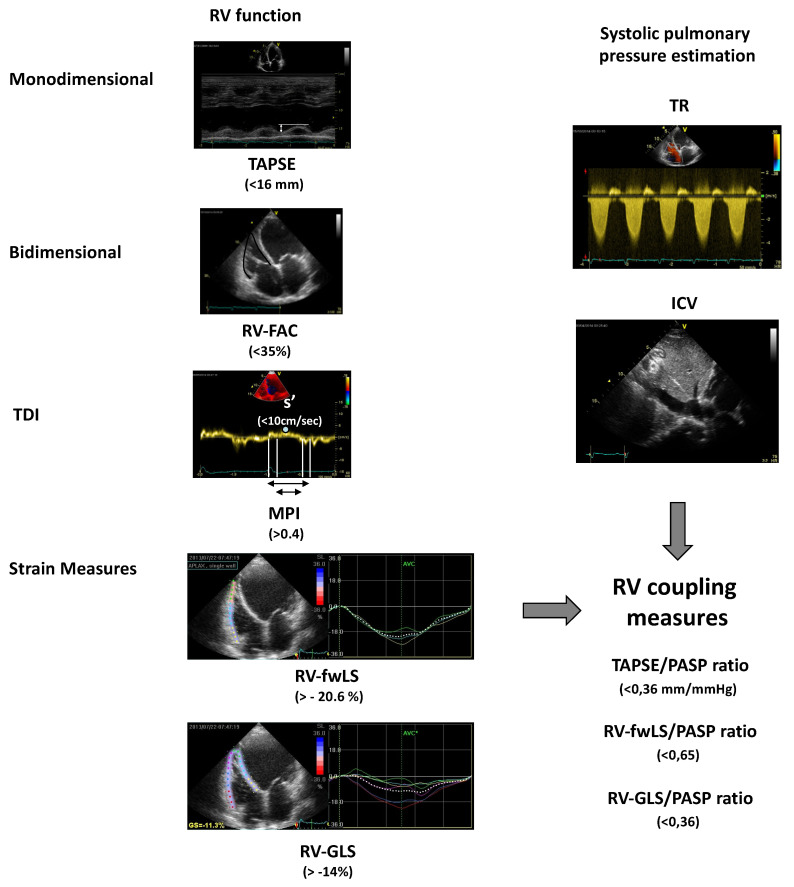
Echocardiographic measures of right ventricle function and right ventriculo-arterial coupling. Between brackets, the values associated with a worse prognosis are reported. ICV: inferior cava vein; MPI: myocardial performance index; PASP: estimated Pulmonary Arterial Systolic Pressure; RV: right ventricle; RV-FAC: right ventricle fractional area change; RV-GLS: RV global longitudinal strain; RV-fwLS: RV free wall longitudinal strain; s’: tricuspid systolic peak at TDI; TAPSE: tricuspid annular plane systolic excursion; TDI: Tissue Doppler Imaging; TR: tricuspid regurgitation.

Figure 2 summarizes the echocardiographic parameters for the assessment of RV function and RV ventriculo-arterial coupling.

### 4.4. RV Function, Central Venous Pressure, and Renal Congestion

In cardiovascular disease, renal function is often impaired. Moreover, the presence of renal dysfunction and its worsening are associated with a poor prognosis [42,43]. From a pathophysiological point of view, the increased central venous pressure (CVP), due to RHF, together with the low cardiac output, plays a key role in the worsening of renal function both in acute and chronic HF [44,45]. From the 1930s, as already shown in HF experimental studies, elevated CVP causes reduced sodium excretion and reduced urine output [46]. This effect is mainly related to the increase in the efferent artery and glomerular capillary pressure, which determines a reduction in the net filtration pressure and, consequently, a fall in the glomerular filtration rate [47].

**Figure 3:**
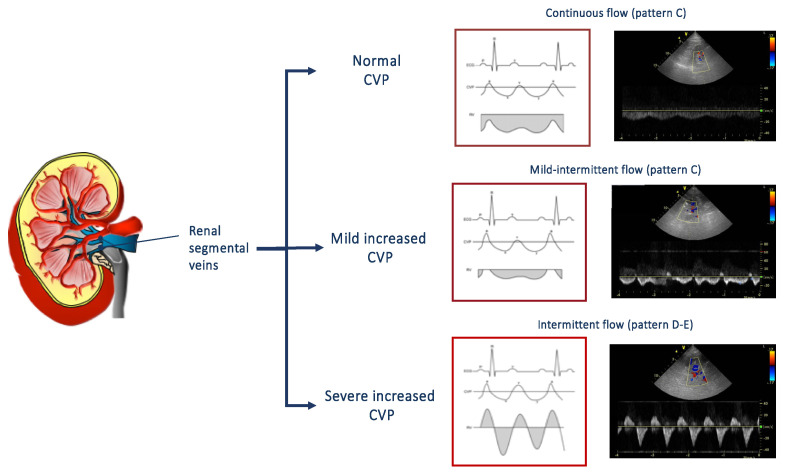
On the right, renal venous patterns evaluated by means of pulsed Doppler at the level of segmental renal veins. In presence of normal central venous pressure, a continuous flow can be observed (top). A mild intermittent (middle) or an intermittent pat-tern (bottom) can be observed according to the presence of a mild or severe increase of central venous pressure. CVP: central venous pressure.

The echocardiographic estimation of CVP, based on the measurement of the diameter and the collapsibility of the vena cava, allows to predict worsening renal function and its related worse outcome [45]. However, CVP is only a surrogate for renal congestion and intrarenal hemodynamic. For this reason, the evaluation of renal venous flow by Doppler ultrasonography has been proposed as a useful tool in order to evaluate the presence of renal congestion [48,49,50]. Renal venous flow is normally continuous, but, in presence of an increased CVP and interstitial renal pressure, it may become intermittent [49]. As shown in Figure 3, the flow patterns, recorded at the level of interlobular renal right veins in the tele-expiratory phase, could be distinguished in continuous, mild-intermittent, and intermittent. An intermittent pattern reflects the effect of increased CVP on the renal flow, i.e., the higher the central venous, the worse the renal venous flow. Interestingly, even the presence of mild-intermittent pattern is independently associated with a greater risk of heart failure progression [50].

### 4.5. Cardiac Magnetic Resonance

Among the diagnostic tools for the study of cardiac structure and function, CMR plays a key role in RV evaluation. CMR is a tomographic technique that allows to accurately evaluate the RV, providing the assessment of RV volumes, mass, systolic function, and myocardial tissue characterization. It has the great advantage of not relying on geometric assumptions. For these reasons, it actually represents the gold standard for the assessment of RV anatomy and function [51]. The standard CMR protocol for the RV evaluation includes balanced steady-state free-precession (SSFP) sequences for cine images, “black blood” T2-weighted (T2W) sequences for the detection of myocardial edema, and contrast enhanced T1-weighted inversion-recovery gradient echo sequences for the assessment of late gadolinium enhancement (LGE-T1W) [52]. Furthermore, velocity-encoded phase-contrast sequences allow to evaluate the flows through stenotic or regurgitant valves, intracardiac shunts, native vessels, and surgically placed conduits [53]. The study of RV systolic function requires SSFP cine imaging. Tracing both endocardial and epicardial borders in a stack of short axis contiguous 5–10 mm thick slices, it is possible to easily calculate RV volumes, mass and EF.

CMR can also provide advanced tissue characterization imaging of the RV myocardium and allows to detect signs of inflammatory activity, i.e., in myocarditis involving the RV. Increased intensity in T2W imaging reflects global or regional myocardial edema [54,55]. Early enhancement after gadolinium in T1W sequences is related to myocardial inflammation, due to a combination of hyperemia, slow washout, and leakage through necrotic cells [56]. The RV diseases could also be associated with a replacement of normal myocardial cells with fibrosis or fat cells, e.g., arrhythmogenic right ventricular cardiomyopathy/dysplasia (ARVC/D), whose histological characteristic is the fibrofatty replacement of the normal myocardium. “Black blood” T1W sequences allows the visualization of the areas of fatty replacement [57,58].

Nevertheless, economic costs and reduced availability prevent the widespread use of CMR as a first line methodology in HF and slow acquisition time and repeated breath holds may limit its feasibility in patients with advanced HF. Finally, implantable devices have traditionally been considered as an obstacle for both safety and image quality. Recent reports suggest that, even if compatible devices related adverse events are infrequent, safety cannot be absolutely guaranteed [59].

## 5. Therapeutic Approaches and Management of Right Ventricular Dysfunction in Chronic Heart Failure

To date, among current HF therapies, there are no RV targeted treatments. The origin and the setting in which RV failure (RVF) occurs should always take into account its management. In patients with HFrEF, the use of neuro-hormonal antagonists (ACE-inhibitors, Angiotensin II Receptor Blockers, Sacubitril–Valsartan, Mineralocorticoid antagonists, and Beta-blockers) are recommended, unless contraindicated or not tolerated, due to their beneficial effect on survival [60]. In RVF, there is also an “iper-activation” of the sympathetic nervous system (SNS) and the renin-angiotensin-aldosterone system (RAAS), but the use of SNS and RAAS inhibitors in RV dysfunction is controversial and lacks clinical evidences.

### 5.1. RAAS Inhibition

In patients with hypoxic pulmonary hypertension (PH) and PH-induced right HF, increased renin activity and elevated aldosterone plasmatic levels have been demonstrated [61]. In these setting, RAAS activation plays an important role in pulmonary vasculature remodeling and pulmonary vasoconstriction. Preclinical studies, that used different models of PH and right HF, showed that ACE inhibitors reduce RV remodeling and improve cardiac function and survival [62,63]. In a small study on 17 patients with systolic left ventricular disease and restrictive cardiomyopathy, Henein et al. found that treatment with ACE-I caused a normalization of right ventricular filling and regression of the restrictive filling on the LV [64]. In spite of this, in a small-randomized placebo-controlled study [65], one-year treatment with ramipril, failed to improve right ventricular function or attenuate remodeling in adult patients with systemic right ventricle. In a multicenter randomized placebo-controlled clinical trial [66], losartan did not seem to improve exercise capacity in adults with systemic right ventricle. Finally, in a double-blind randomized placebo-controlled pilot trial [67], neither RVEF, exercise capacity, nor quality of life, improved with valsartan.

### 5.2. Beta-Blockers

There are few studies about the effects of beta-blockers on RVF. Tatli et al. [68] found a significant increase in RV systolic performance in patients with HF after treatment with carvedilol, associated with an improvement of LV systolic performance. In another small cohort study [69], carvedilol treatment increased RVEF and decreased RV end-systolic and end-diastolic volume in patients with systemic right ventricle. Nonetheless, in a small series of patients with porto-pulmonary hypertension, Provencher et al. [70] reported significant functional improvement after two months of beta-blocker withdrawal. These results may be explained with the removal of the negative chronotropic effect of β-blockers in pulmonary hypertensive patients. In fact, in these sets of patients, there is a limited increase in stroke volume and the inability to increase heart rate during exercise. In this context, cardiac output increases with the rise of heart rate.

In conclusion, well-established therapy of HFrEF could be relevant for RVF as well. However, in PH, the use of ACE inhibitors, angiotensin II blockers, and selective beta-blockade have not been explored, and clinical investigations are needed.

### 5.3. Volume Management

A small amount of intravenous volume administration may be appropriate in hypotensive patients with RVF and without increased CVP, as in case of right ventricular myocardial infarction [71]. On the other hand, in RVF with increased CVP the management of volume overload plays a key role in acute and chronic patients. It is generally based on salt and fluid restriction, administration of loop diuretics, and renal replacement therapy in the case of refractoriness to medical therapy. In patients with acute decompensated HF, RV unloading increases RV contractility, cardiac output, and renal perfusion in acute setting. In CHF patients with RVF, volume overload is associated with worse outcomes and a diuretic approach should be adopted to reduce CVP.

### 5.4. Pressure Overload: Pulmonary Hypertension

Pulmonary hypertension (PH) due to left heart disease (LHD) is the most common cause of RV dysfunction. It is characterized by an increase in mean pulmonary artery pressure over 25 mmHg, a pulmonary artery wedge pressure (PAWP) > 15 mmHg, and a normal or reduced cardiac output [72]. In HF due to PH, we can find two major components: hydrostatic and vasoreactive. The backward transmission of elevated left ventricular end-diastolic pressure is the hydrostatic component; the vasoreactive one develops with longstanding increase in pulmonary artery pressure and it is characterized by vasospasm, vasoconstriction, and changes in pulmonary vasculature [73].

The (global) management of underlying condition and co-morbidities should be the primary aim of PH-LHD therapy. Then, specific measures to treat PH, such as repair of valvular heart disease and aggressive therapy for HF with reduced systolic function, should be taken. Endothelial dysfunction has been proposed as a cause of PH and, hence, as a target for treatment in these patients, but therapies directed to the pulmonary vasculature are currently not approved in PH-LHD [74]. Among the promising novel therapeutic approach, Sildenafil [75,76], a selective inhibitor of type 5 phosphodiesterase, seems to provide favorable effects on hemodynamic and oxygen uptake in acute and chronic settings. Riociguat [77], a soluble guanylate cyclase stimulator, seems to improve cardiac function and pulmonary systemic vascular resistance. It is currently under investigation as a therapy for PH.

### 5.5. Right Ventricular Function and Resynchronization Therapy

Cardiac resynchronization therapy (CRT) is an established treatment that can be used in patients with HFrEF and intraventricular conduction delay [60] in order to improve quality of life and mortality in symptomatic patients with HFrEF. The benefit of patients with right sided HF from CRT is still debated. In patients with left ventricular reverse remodeling, CRT seems able to, also, induce RV reverse remodeling and improvement of RV systolic function [78]. This result is partly due to CRT-induced volumetric chambers reduction with consequent improvement of mitral regurgitation and pulmonary vein hypertension and with an additional positive effect on the mechanics of the inter-ventricular septum. Moreover, RV coupling to the pulmonary arterial circulation (RV-PA coupling) is associated with a greater LV remodeling after CRT [79].

### 5.6. Right Ventricular Function and Left Ventricular Assistance Device

Although this argument is beyond the purpose of this review, RV evaluation has a key role in the selection of candidates to left ventricle assist devices (LVADs) implantation. Patients with advanced heart failure may benefit from mechanical circulatory support as a bridge to heart transplantation, bridge to decision, or as destination therapy. When assessing treatment options for advanced HF, right HF (RHF) needs to be evaluated for adequately planning the best treatment solution. The presence of a preserved right ventricular function can, in fact, minimize the occurrence of RHF, which ranges between 10% and 40% after LVAD implantation [80,81,82]. It is due to several mechanisms: augmentation of cardiac output by the increase amount of venous return to the right ventricle, altered geometry with septal shift towards the left ventricular cavity due to unloading by the device, and an increase in pulmonary vascular resistance encountered in the perioperative period.

All previously discussed echocardiographic indices may be useful to assess RV function and should be considered together with clinical, hemodynamic, and biochemical parameters for predicting right heart failure [83]. However, the lack of a uniform definition of RHF, the retrospective setting of the principle studies, and the single-center small populations examined, probably make the evaluation of a reliable score very challenging [84,85,86,87,88,89]. In a recent meta-analysis [90], several parameters (clinical, biochemical, hemodynamic, and echocardiographic) have been evaluated in 4428 patients, but none of these were able to accurately identify patients at risk for RHF. In this setting, RV longitudinal strain may be very useful, preoperatively, in predicting RHF after LVAD implantation [91], but more extensive multicenter studies are needed to fully validate this parameter. Finally, TAPSE/PASP ratio, as a surrogate of RV-PA coupling, seems to be not predicting of early RHF post LVAD implantation, even if a higher ratio was associated with the need for a right ventricular assist device (RVAD) [92].

To date, even if cardiac magnetic resonance represents the gold standard for determining right heart size and function, the practical use is limited by its availability and the compatibility with several cardiac devices.

### 5.7. Right Ventricular Mechanical Circulatory Support

In advanced HF with severe right ventricular dysfunction, right ventricular assist devices (RVADs) have also been proposed. For most people, RVAD is a temporary solution in acute RHF secondary to several diseases or to treat RHF after LVAD [93,94]. Percutaneous options (such as the Impella RP and the TandemHeart RVAD with the PROTEK Duo cannula) have been introduced for temporary RV support [95]. For long-term RV support, a biventricular assist device or total artificial heart are the only available strategies [96], whereas the off-label use of LVAD for the right-sided circulation is limited by the different anatomy, and physiology of RV and pulmonary circulation [97].

## 6. Conclusions

RV failure has a significant clinical and prognostic impact in HF patients. Assessment of RV structure and function has traditionally been difficult when compared with LV study due to particular RV position and shape. The introduction of new echocardiographic techniques and of CMR represents a big and significant step forward in the improvement of diagnosis of RV failure. It is recommended to integrate clinical evaluation with imaging techniques in order to better characterize RV and HF phenotype.

The therapeutic management of RV failure is still a challenge in both acute and chronic settings. Pharmacological treatment, with proven effectiveness in LV HF, has not demonstrated the same efficacy in RV HF, and has not been studied for left-side HF. An integrated approach, including pharmacological and device treatment, could be useful in improving symptoms and prognosis. Big efforts are needed for the study and research of better target molecules and devices, as well as more effective treatments of RV dysfunction.
